# Supplementary material for: Comprehensive sequencing of the lung neuroimmune landscape in response to asthmatic induction
Source: Front Immunol. 2025 Mar 13;16:1518771. doi: 10.3389/fimmu.2025.1518771 (PMC11965707; doi:10.3389/fimmu.2025.1518771)
Supplement: Supplementary file 5 [file Presentation1.pdf]

Supplementary Figure 1.

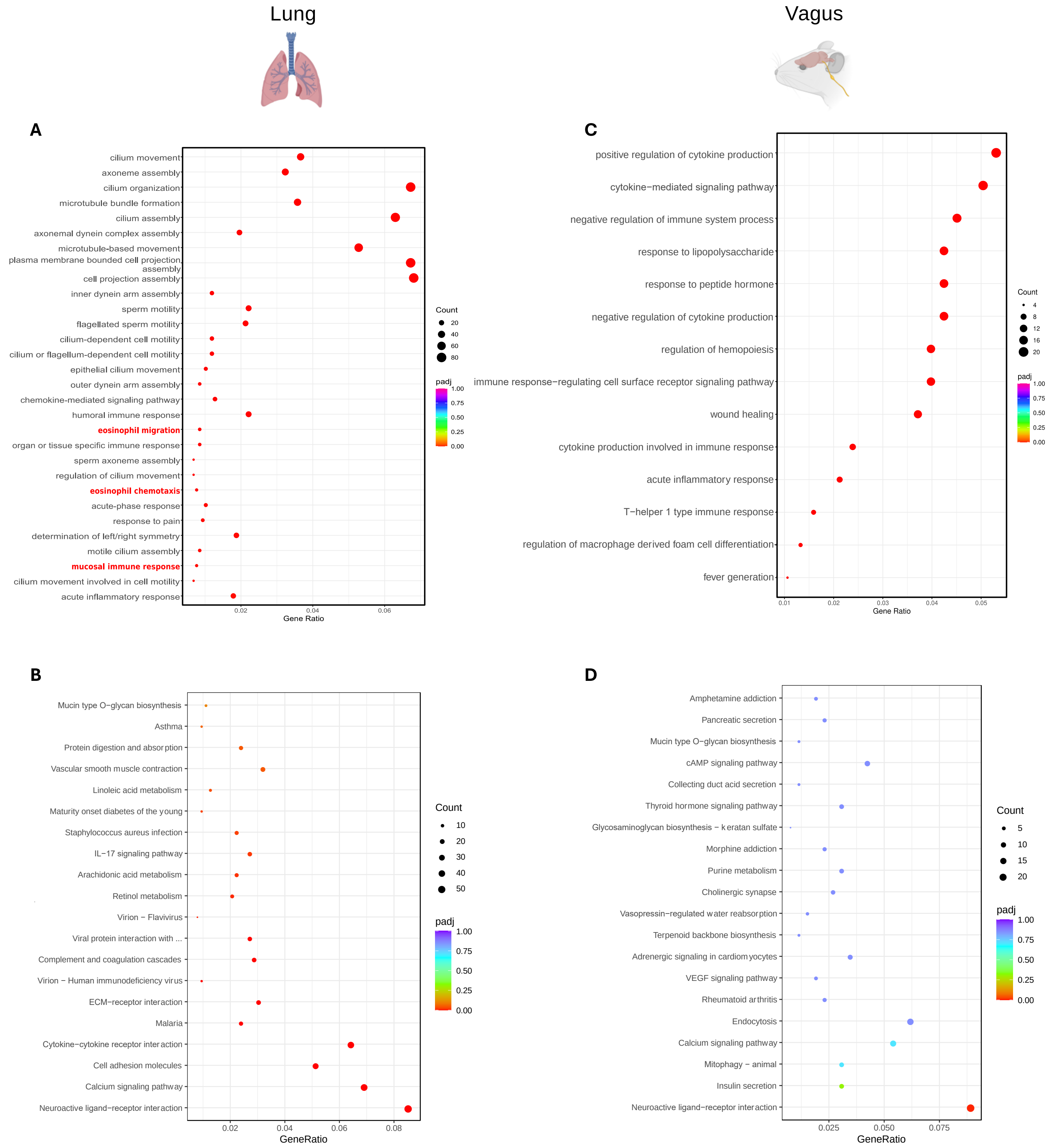

Supplementary figure 1. GO and KEGG analysis. LUNG: A) GO dot plot showing enriched processes differentiating *A. alternata*-induced asthma conditions from control. **B)** KEGG dot plot highlighting pathway enrichment differences between *A. alternata*-induced asthma from control. **C)** GO dot plot showing enriched processes differentiating *A. alternata*-induced asthma conditions from control. **D** KEGG dot plot highlighting pathway enrichment differences between *A. alternata*-induced asthma from control.

Supplementary Figure 2.

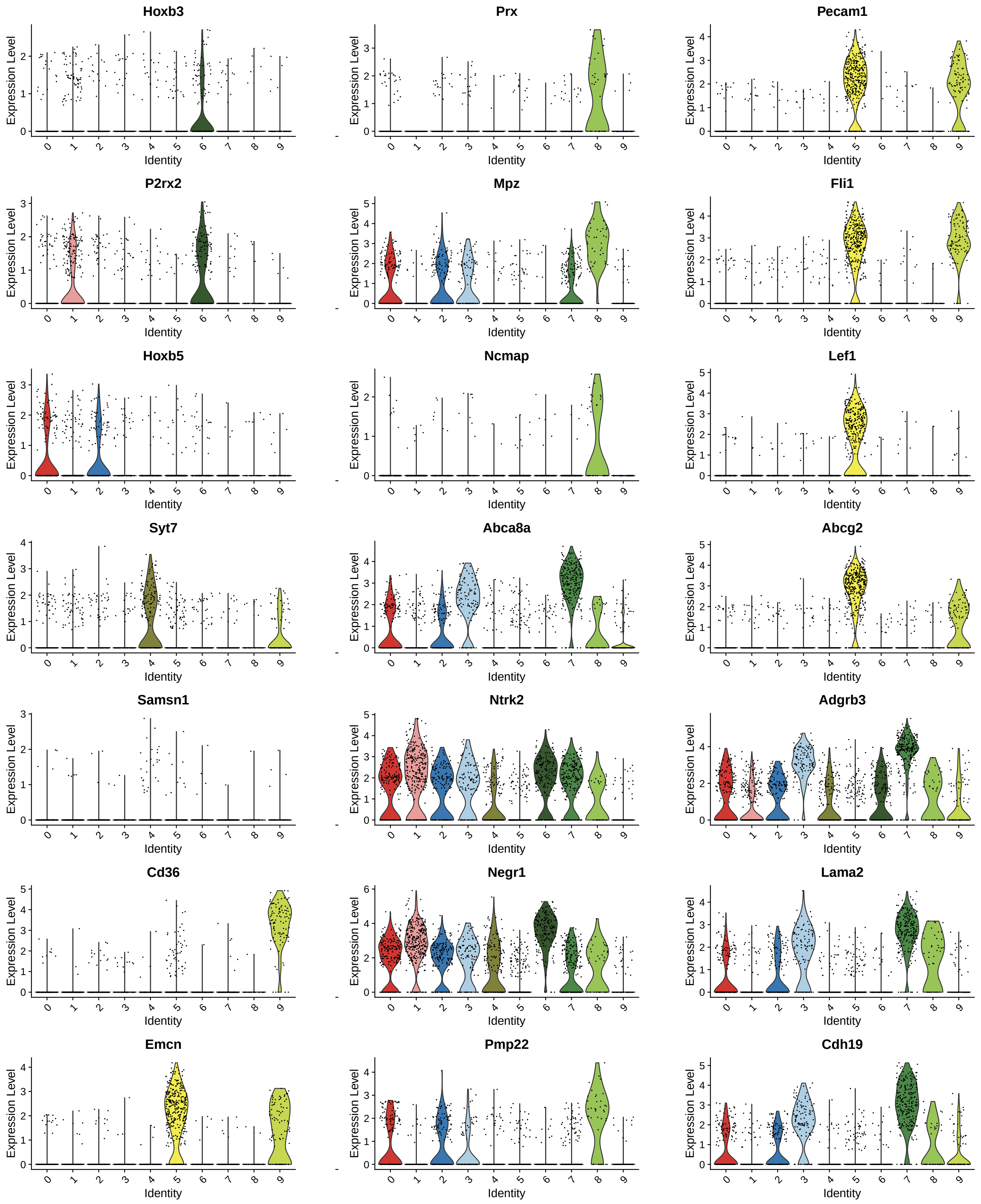

Supplementary figure 2. Canonical gene markers identifying cell types in single-nuclei RNA-seq dataset a) Violin plots showing the expression of key marker genes in the 11 unbiased clusters.

Supplementary Figure 3.

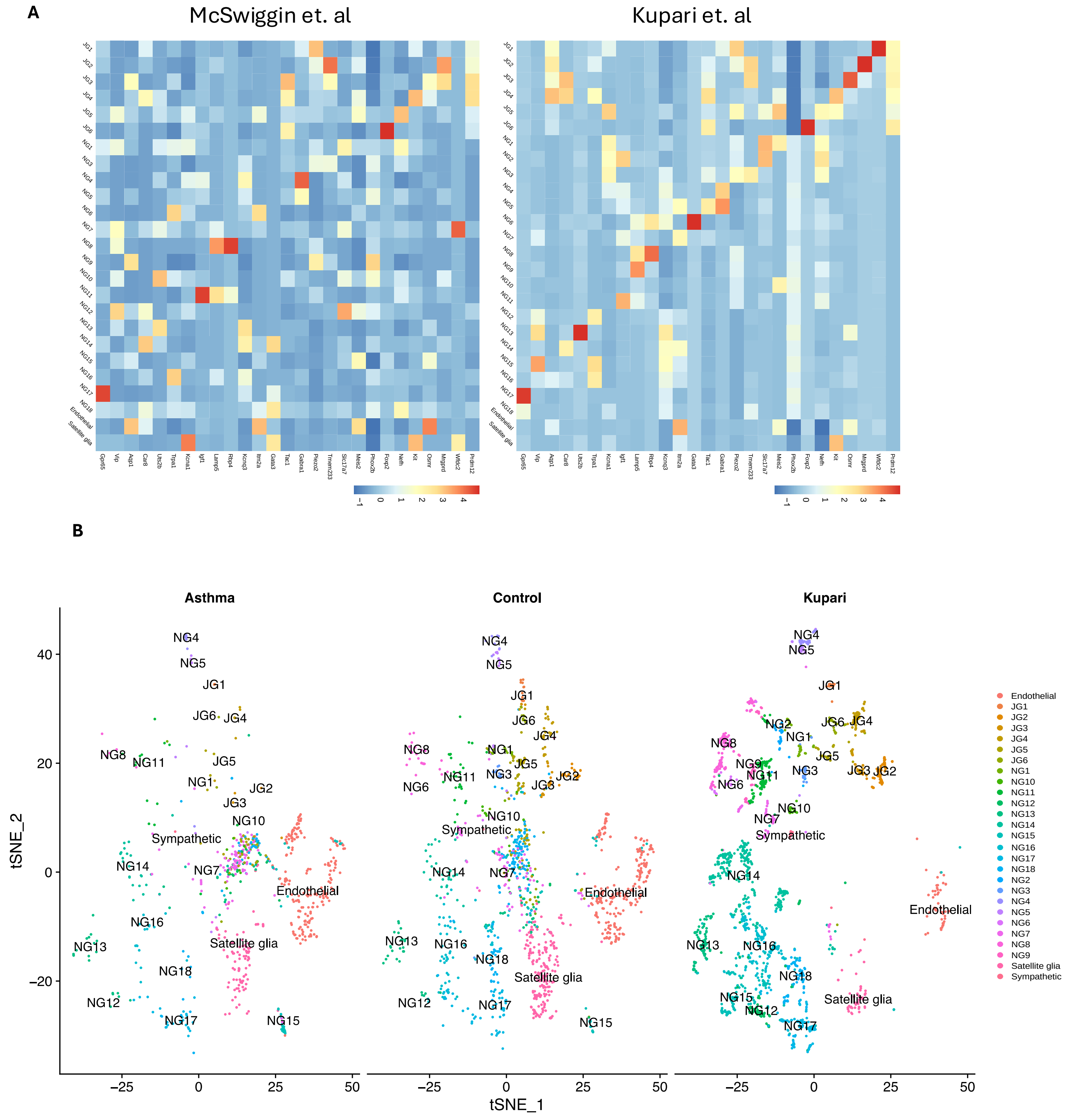

Supplementary figure 3. Single-nucleus sequencing of control and asthmatic vagal ganglia are similar to previous single-cell sequencing of healthy vagal ganglia. A) Heatmap of showing expression of key genes from the data provided by Kupari et al. in the current dataset **B**) Tsne reveals a high similarity between the current single-nucleus analysis to the single cell analysis of Kupari et al.

Supplementary Figure 4.

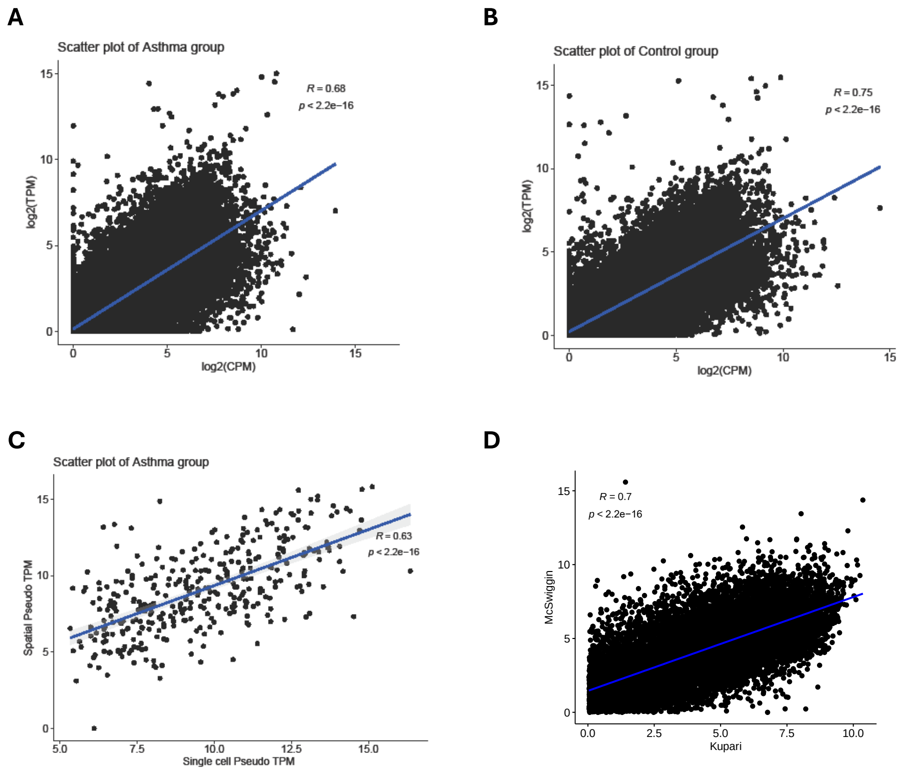

Supplementary figure 4. Correlation between datasets. A) Correlation between bulk and single nucleus sequencing of vagal transcripts in *A. alternata*. **B**) Correlation between bulk and single nucleus sequencing of vagal transcripts in control. **C**) Correlation between single nucleus sequencing and spatial sequencing of vagal transcripts. **D)** Correlation between the current single-nucleus dataset and previously published single-cell dataset from Kupari *et. al*.

Supplementary Figure 5.

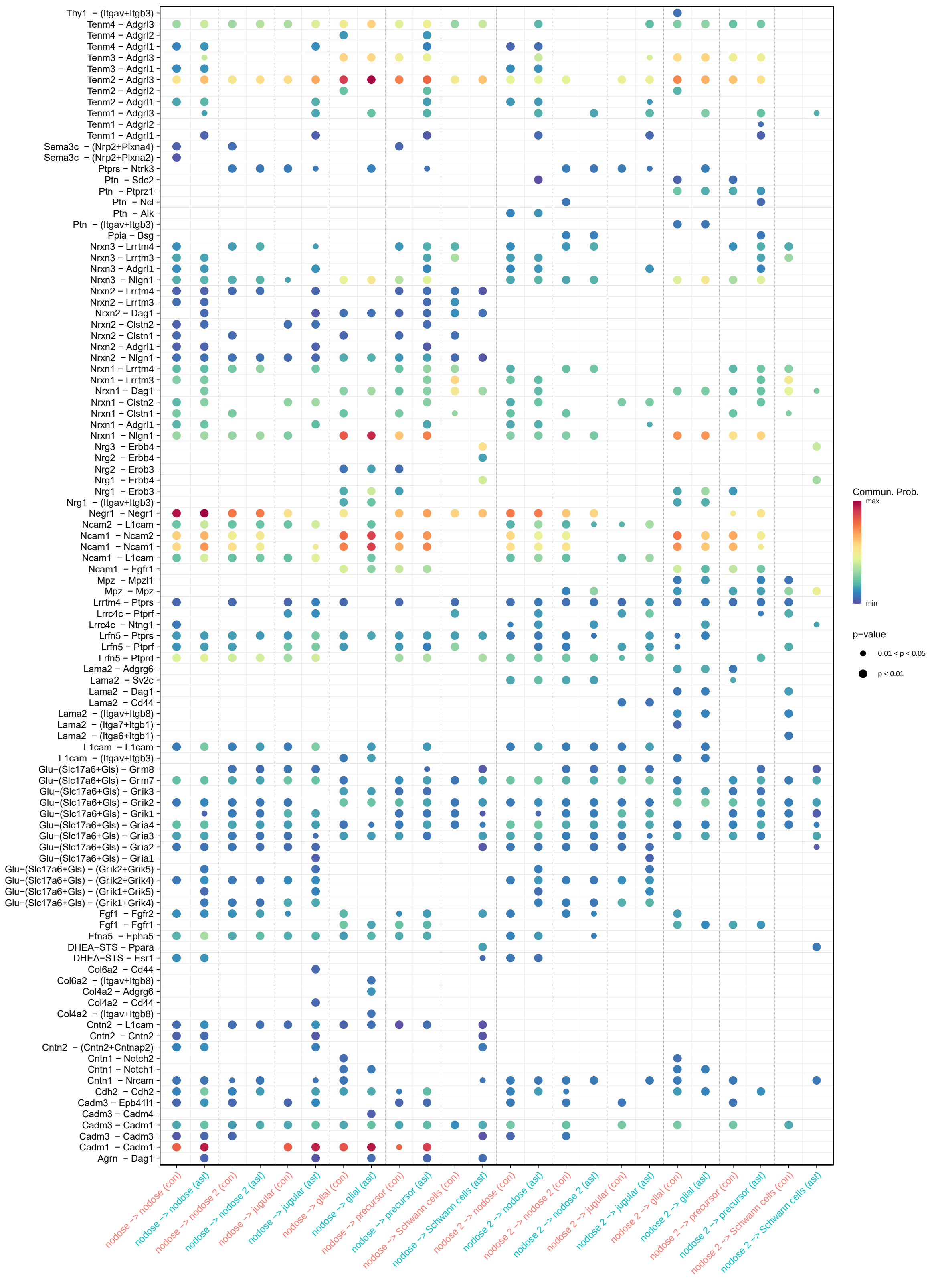

Supplementary figure 5. Differential CellChat expression. Differential expression of ligands and receptors between *A. alternata* and control vagal transcripts. Jugular was the source and all other cell types were targets.
